# Thickness of retinal layers in the foveas of children with anisometropic amblyopia

**DOI:** 10.1371/journal.pone.0174537

**Published:** 2017-03-22

**Authors:** Wuhe Chen, Jinling Xu, Jinjing Zhou, Zhouqun Gu, Shenghai Huang, Heming Li, Zhuoer Qin, Xinping Yu

**Affiliations:** School of Ophthalmology and Optometry and Eye Hospital of Wenzhou Medical University, Wenzhou, Zhejiang, China; Bascom Palmer Eye Institute, UNITED STATES

## Abstract

**Purpose:**

To use highly precise spectral-domain optical coherence tomography (SD-OCT) to determine whether there were structural abnormalities in the layers of different regions of the fovea in children with anisometropic amblyopia.

**Methods:**

Eighteen children (mean age 7.8 years old; range 5–11 years) with unilateral anisometropic amblyopia and 18 age-matched control subjects participated. Foveal thickness was measured with an enhanced depth imaging system, SD-OCT and segmented into layers using custom developed software. The thickness of each layer of the fovea was compared among amblyopic eyes, fellow eyes and control eyes with optical magnification correction for axial length and statistical correction for age and sex.

**Results:**

The total thickness and each intra-ocular layer of the central fovea were the same for each group. However, the amblyopic eyes were significantly thicker than the normal control eyes in 2 of 4 quadrants of the peripheral retina. Exploring intra-retinal layers in these two quadrants, the nasal nerve fiber layer (NFL) and inferior inner nuclear layer (INL)were significantly thicker in amblyopic eyes than in control eyes (p = 0.01 and 0.012, respectively, by ANCOVA).

**Conclusion:**

The SD-OCT data revealed marginal differences in some foveal layers at peripheral locations and indicated that structural differences might exist between individuals with amblyopia and visually normal control subjects. However, the differences were scattered and represented no identifiable pattern. More studies with large samples and precise locations of the retinal layers must be performed to extend the present results.

## Introduction

Amblyopia is the most common cause of reduced vision in children, affecting approximately1.6% to 3.6% of the population.[1] It can be caused by disruption of binocular vision during the period of neural plasticity early in life, such as due to strabismus, refractive error differences, or visual form deprivation. When strabismus and/or anisometropia are present in the eyes, normal binocular integration cannot occur. One eye loses the competition between the two eyes in the abnormal interocular system, and unilateral amblyopia occurs. Extracellular recordings from the striate cortex neurons of kittens and monkeys with strabismic and anisometropic amblyopia have shown that most striate cortex neurons respond to the sound eye.[2,3]In addition to cortical deficits, cells in the lateral geniculate nucleus layers of amblyopic eyes were less developed than those in the dominant eye’s layers.[4,5]These findings indicated that functional changes involved in amblyopic development could occur at various levels of the visual pathway. However, whether the earliest site of the visual pathway, the retina, is involved remains a mystery.

The majority of previous animal and human studies have failed to show retinal abnormalities in amblyopia.[6–9]However, among a large number of retinal thickness studies using OCT, recent results have been inconsistent. Earlier, with time-domain OCT (TD-OCT), several studies showed that there was no difference in macular thicknesses in the foveas of children with unilateral amblyopia,[10–12]while other studies showed a thicker fovea in unilateral amblyopia[13,14] or a thicker peripapillary retinal nerve fiber layer.[15]

Later, with the development of spectral-domain OCT (SD-OCT), researchers started to evaluate the thickness of each layer of the fovea in amblyopia. To date, the literature has shown conflicting results.[15–18] For example, Nishi et al.[16] detected that the length of the photoreceptor outer segment (OS) layer in the fovea was significantly thinner in anisohypermetropic amblyopic eyes than in fellow eyes. Al-Haddad et al.[17] also noted decreased length of the central foveal cones in amblyopia eyes, as well as a thicker overall fovea. Park et al.[18] found a notable difference between amblyopic and fellow eyes in both the ganglion cell layer and the inner plexiform layer but not in the OS layer. Bruce et al.[19] found no difference between amblyopic and fellow eyes but a significant difference between the eyes of amblyopes and controls. These conflicting findings could result from differences in ethnicity, the age of the subjects, severity of amblyopia, or the methods of measuring the thickness of the foveal layers. Thus, the purpose of this study was to determine the thickness of each retinal layer in different regions of the fovea (within a 0.5mm radius of the foveal center) in the eyes of children with unilateral anisometropic amblyopia and to compare the findings with those from the fellow eyes and the eyes of age-matched controls. The null hypothesis was that retinal layer thicknesses in the foveas of amblyopic eyes would not differ from those of the fellow eyes or the control subjects’ eyes.

## Materials and methods

### Subjects

This was a cross-sectional, comparative, prospective study conducted at the Wenzhou Medical University Affiliated Eye Hospital between August 2013 and November 2014. The protocol of the study was approved by the Review Board of Wenzhou Medical University and was performed according to the tenets of the Declaration of Helsinki for research involving human subjects. Prior to enrolling the children in the study, the children and parents were informed about the purpose and methods of the study and then were provided with an informed consent agreement to sign. The signed informed consent forms were returned to the researcher before the examinations were performed on the children.

Children with the diagnosis of unilateral amblyopia and visually normal control subjects who were able to cooperate sufficiently to participate in the OCT examination were prospectively recruited. Amblyopia was defined based on the Preferred Practice Pattern (PPP) [20]: unilateral amblyopia > = 2 line interocular difference, with the presence of anisometropia. Anisometropia was defined as an interocular difference in refraction (spherical equivalent) of more than 1.0 diopter (D) and no manifest squint.[21]The inclusion criteria were children with unilateral amblyopia caused by hyperopic anisometropia at between 5 and 12 years of age, who had central fixation as determined by ophthalmoscopy. An equivalent group of age-matched controls without amblyopia were also included. The control group was composed of children whose BCVA was equal to or better than 20/20, and children with myopia greater than –0.50 D were excluded. Patients with neurological diseases, ocular conditions such as glaucoma or retinal disorders, and nystagmus were excluded from the study. All of the patients and controls underwent comprehensive eye examinations, including VA, retinoscopy after pupillary dilation, slit-lamp examination, fundoscopy and an orthoptic evaluation, including Hirschberg testing, cover testing, and extra-ocular motility assessment. The IOL-Master (version 5.0; Carl Zeiss, Jena, Germany) was used to measure axial length. The thickness of the retinal fovea was measured using SD-OCT through a natural pupil.

### Procedures

The foveal thicknesses of all of the patients and controls were examined using an enhanced depth imaging (EDI) system (Spectralis OCT; Heidelberg Engineering, Heidelberg, Germany; wavelength: 870 nm; scan pattern: enhanced depth imaging), which was reported previously.[22]The system is an SD-OCT that has eye tracking and is capable of up to 100 separate OCT scans at any arbitrary location, and it can produce high resolution images. The right eye was studied first, followed by the left eye. The center of each volumetric measurement was adjusted to be the foveal center. We identified the fovea at the center of the vessel-free area during the recordings of the images. The diameter of this area is approximately 1mm. Each subject’s horizontal and vertical scans with the highest quality containing the center of the fovea were selected for evaluation. Image selection was based on a subjective assessment of the image resolution and retinal architecture.

**Fig 1:**
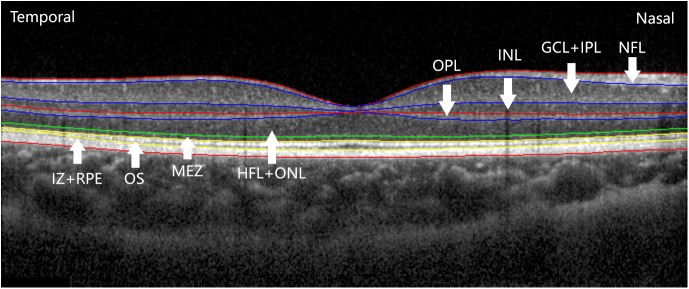
Intra-retinal layers on SD-OCT macular images. This image, obtained at the horizontal meridian of the right eye, shows eight intra-retinal layers. Images obtained at the vertical meridian by SD-OCT were similar to this one, except that the horizontal image showed asymmetry between the nasal and temporal locations.

Custom software for automatic segmentation into layers was developed to measure the thicknesses of eight intra-retinal layers seen on the 2-D images produced by the OCT instrument.[23]Nine boundaries between the intra-retinal layer structures were detected, and the thickness profiles of eight intra-retinal layers were determined: (1) nerve fiber layer (NFL); (2) ganglion cell layer and inner plexiform layer (GCL+IPL); (3) inner nuclear layer (INL); (4) outer plexiform layer (OPL); (5) Henle fiber layer and outer nuclear layer (HFL+ONL); (6) myoid and ellipsoid zone (MEZ); (7) outer segment (OS) of receptors; and (8) interdigitation zone and retinal pigment epithelium/Bruch’s complex (IZ+RPE) (Fig 1). The distances between each boundary were measured in microns and were automatically transferred to a Microsoft Excel file. On the OCT images, the center of the fovea was where the inner retinal layers (the nerve fiber layer, ganglion cell layer and inner plexiform layer, inner nuclear layer, outer plexiform layer) were absent. The thicknesses of four layers (HFL+ONL, MEZ, OS, IZ+RPE) in the central fovea and eight layers (NFL, GCL+IPL, INL, OPL, HFL+ONL, MEZ, OS, IZ+RPE) in the peripheral fovea were chosen for analysis. The thickness at the foveal center was defined as the mean value obtained at the foveal center from the horizontal and vertical scans. The thickness of the peripheral fovea was defined as locations 0.5mm from the foveal center in the superior, inferior, nasal and temporal directions. All of the detected intra-retinal layer boundaries were segmented using graph theory and the shortest-path search method, based on an optimization algorithm of the dynamic programming technique. We obtained thickness profiles of the 8 intra-retinal layers after adjusting the ocular magnification with Bennett’s formula[24,25], which was described in an earlier article published by our cooperative group.[23]

### Statistical analysis

Statistical analysis was performed using SPSS software (version 19.0; SPSS Inc, Chicago, Illinois, USA). The data of the left eyes of control subjects were used for analysis because the image quality was superior to that of the right eyes. Visual acuity data were converted into the logarithm of the minimal angle of resolution (logMAR) for statistical calculation and analysis. Refraction data were converted into SEs, which were calculated as the spherical dioptric power plus one-half of the cylindrical dioptric power. Multivariate analysis of covariance (ANCOVA) was used to evaluate differences among amblyopic eyes, fellow eyes and normal control eyes while adjusting for the possible effects of age and sex. After ANCOVA, the differences among the amblyopic eyes, its fellow eyes and control eyes were examined by Bonferroni’s test. The t test was used to compare spherical equivalent (SE) refractive errors, axial length (AL) and BCVA among the groups. Associations between macular thicknesses and the AL, SE and VA differences were determined with Pearson’s correlation tests. P values <0.05 were considered to be statistically significant.

## Results

### Subject data

Eighteen patients (7.8±1.9 years old; boys/girls, 14/4) with unilateral amblyopia and 18 age-matched controls (7.7±1.0 years old, boys/girls; 11/8) were included. Four strabismic, amblyopic subjects were excluded because there were too few data for meaningful statistical analysis, and we considered that dissimilar amblyopias could have differing impacts on the macula.[26–28]The cause of amblyopia was identified as anisometropia in all of the included subjects. There was no significant difference in age between the amblyopic and control groups (p = 0.915, t-test).

Refraction, axial length and VA status are presented in Table 1. The amblyopic eyes were significantly more hyperopic than the fellow eyes and control eyes (p<0.001, t-test). The axial length of the amblyopic eyes was significantly shorter than in the other two groups (p<0.001, t-test). The amblyopic eyes had significantly worse BCVA than the fellow eyes and control eyes (p<0.001, t-test).The BCVA difference between the two eyes of the amblyopic subjects ranged from 2.2 to 10 Snellen lines.

**Table 1:** Baseline characteristics of amblyopic subjects participating in the study.

	*Refraction (D)*	*Axial length (mm)*	*VA (logMAR)*
*Amblyopic eyes (n = 18)*	*+4*.*44±1*.*57*	*21*.*77±0*.*68*	*0*.*48±0*.*19*
	*(+2*.*00-+7*.*63)*	*(20*.*52–23*.*32)*	*(0*.*22–1*.*00)*
*Fellow eyes (n = 18)*	*+0*.*75±0*.*98*	*23*.*17±0*.*75*	*0*.*00±0*.*01*
	*(-0*.*25-+3*.*75)*	*(21*.*75–24*.*41)*	*(0*.*00–0*.*05)*
*Control eyes (n = 18)*	*0*.*00±0*.*29*	*22*.*97±0*.*69*	*0*.*01±0*.*03*
	*(-0*.*50-+0*.*50)*	*(21*.*85–24*.*33)*	*(-0*.*08–0*.*05)*

### Intra-retinal thickness in the central fovea

The retinal layer thickness data presented here were adjusted for the ocular magnification factor and were statistically corrected for age and sex by ANCOVA. The resultant p values were corrected by Bonferroni’s correction due to multiple testing. (Table 2). Total central foveal thickness was 133.58±8.16μm in the amblyopic eyes, 132.85±9.48μm in the fellow eyes and 133.31±6.31μm in the control eyes (p>0.999, ANCOVA). The mean HFL+ONL, MEZ, OS and IZ+RPE thicknesses in the central fovea in amblyopic eyes were 65.11±8.19μm, 16.80±1.15μm, 26.56±1.13μm and 25.09±1.83μm, respectively. The mean thicknesses of the same layers for the fellow eyes were 64.46±9.11μm, 17.11±1.73μm, 26.02±1.04μm and 25.25±1.52μm, respectively. Further, the mean thicknesses of the same layer in the control eyes were 66.58±6.49μm, 15.96±0.85μm, 25.86±0.92μm and 24.90±1.76μm, respectively. Only MEZ showed a significant difference between the fellow eyes and control eyes (p = 0.035).

**Table 2:** Comparisons of each retinal layer thickness in amblyopic, fellow and control eyes with correction for ocular magnification, age and sex.

*Retinal layers*	*Amblyopic*	*Fellow*	*Control*	*p-value*	*p-value*	*p-value*
	*eyes (n = 18)*	*eyes(n = 18)*	*eyes(n = 18)*	*Amblyopic vs fellow*	*Amblyopic vs control*	*fellow vscontrol*
***Central fovea***						
***Total***	***133*.*58±8*.*16***	***132*.*85±9*.*48***	***133*.*31±6*.*31***	*>****0*.*999***	*>****0*.*999***	*>****0*.*999***
*HFL+ONL*	*65*.*11±8*.*19*	*64*.*46±9*.*11*	*66*.*58±6*.*49*	*>0*.*999*	*>0*.*999*	*>0*.*999*
*MEZ*	*16*.*80±1*.*15*	*17*.*11±1*.*73*	*15*.*96±0*.*85*	*>0*.*999*	*0*.*181*	*0*.*035*
*OS*	*26*.*56±1*.*13*	*26*.*02±1*.*04*	*25*.*86±0*.*92*	*0*.*400*	*0*.*167*	*>0*.*999*
*IZ+RPE*	*25*.*09±1*.*83*	*25*.*25±1*.*52*	*24*.*90±1*.*76*	*>0*.*999*	*>0*.*999*	*>0*.*999*
***Peripheral***	***fovea***					
***Nasal***						
***Total***	***224*.*95±9*.*92***	***217*.*78±11*.*43***	***215*.*10±12*.*36***	***0*.*205***	***0*.*041***	*>0*.*999*
*NFL*	*18*.*26±1*.*68*	*17*.*15±1*.*82*	*16*.*38±2*.*31*	*0*.*221*	*0*.*010*	*0*.*633*
*GCL+IPL*	*43*.*16±5*.*67*	*39*.*91±6*.*77*	*38*.*81±7*.*14*	*0*.*464*	*0*.*175*	*>0*.*999*
*INL*	*20*.*08±2*.*55*	*20*.*52±2*.*57*	*20*.*77±3*.*14*	*>0*.*999*	*>0*.*999*	*>0*.*999*
*OPL*	*12*.*76±2*.*65*	*15*.*99±6*.*77*	*12*.*57±3*.*89*	*0*.*143*	*>0*.*999*	*0*.*108*
*HFL+ONL*	*65*.*81±5*.*54*	*60*.*17±8*.*43*	*62*.*63±6*.*00*	*0*.*052*	*0*.*512*	*0*.*863*
*MEZ*	*16*.*29±1*.*01*	*16*.*32±1*.*45*	*16*.*29±1*.*52*	*>0*.*999*	*>0*.*999*	*>0*.*999*
*OS*	*24*.*05±1*.*38*	*23*.*54±1*.*60*	*22*.*84±2*.*00*	*>0*.*999*	*0*.*112*	*0*.*671*
*IZ+RPE*	*24*.*51±1*.*84*	*24*.*15±2*.*33*	*24*.*82±2*.*08*	*>0*.*999*	*>0*.*999*	*>0*.*999*
***Temporal***						
***Total***	***216*.*87±11*.*88***	***213*.*39±10*.*93***	***208*.*22±11*.*15***	*>0*.*999*	***0*.*092***	***0*.*567***
*NFL*	*16*.*93±1*.*60*	*16*.*50±2*.*29*	*15*.*57±2*.*32*	*>0*.*999*	*>0*.*999*	*0*.*583*
*GCL+IPL*	*38*.*25±5*.*57*	*38*.*95±6*.*07*	*36*.*80±6*.*17*	*>0*.*999*	*>0*.*999*	*0*.*887*
*INL*	*19*.*13±3*.*76*	*18*.*75±3*.*18*	*19*.*44±3*.*17*	*>0*.*999*	*>0*.*999*	*>0*.*999*
*OPL*	*20*.*02±10*.*26*	*13*.*74±5*.*04*	*17*.*10±9*.*83*	*0*.*116*	*0*.*983*	*0*.*784*
*HFL+ONL*	*59*.*19±11*.*48*	*61*.*60±6*.*74*	*56*.*02±13*.*56*	*>0*.*999*	*>0*.*999*	*0*.*420*
*MEZ*	*16*.*22±1*.*08*	*16*.*53±0*.*74*	*16*.*52±1*.*28*	*>0*.*999*	*>0*.*999*	*>0*.*999*
*OS*	*23*.*87±2*.*00*	*23*.*32±2*.*17*	*22*.*98±2*.*50*	*>0*.*999*	*0*.*732*	*>0*.*999*
*IZ+RPE*	*24*.*23±3*.*50*	*23*.*97±2*.*95*	*23*.*80±2*.*62*	*>0*.*999*	*>0*.*999*	*>0*.*999*
***Superior***						
***total***	***229*.*06±9*.*45***	***221*.*44±14*.*40***	***221*.*17±10*.*71***	***0*.*189***	***0*.*236***	*>0*.*999*
*NFL*	*16*.*66±3*.*27*	*17*.*77±3*.*75*	*17*.*41±4*.*72*	*>0*.*999*	*>0*.*999*	*>0*.*999*
*GCL+IPL*	*48*.*27±6*.*88*	*47*.*85±6*.*03*	*47*.*15±7*.*05*	*>0*.*999*	*>0*.*999*	*>0*.*999*
*INL*	*25*.*27±3*.*35*	*23*.*93±3*.*12*	*22*.*81±2*.*90*	*0*.*603*	*0*.*070*	*0*.*876*
*OPL*	*22*.*46±8*.*67*	*21*.*37±6*.*61*	*23*.*71±6*.*92*	*>0*.*999*	*>0*.*999*	*>0*.*999*
*HFL+ONL*	*52*.*13±14*.*20*	*46*.*18±13*.*84*	*47*.*52±8*.*39*	*0*.*511*	*0*.*877*	*>0*.*999*
*MEZ*	*16*.*98±2*.*59*	*17*.*67±3*.*63*	*16*.*76±1*.*48*	*>0*.*999*	*>0*.*999*	*>0*.*999*
*OS*	*23*.*38±1*.*54*	*22*.*74±1*.*28*	*22*.*42±3*.*77*	*>0*.*999*	*0*.*776*	*>0*.*999*
*IZ+RPE*	*23*.*88±2*.*56*	*23*.*90±1*.*72*	*23*.*98±3*.*05*	*>0*.*999*	*>0*.*999*	*>0*.*999*
***Inferior***						
***total***	***230*.*76±9*.*86***	***221*.*78±11*.*48***	***219*.*90±12*.*51***	***0*.*076***	***0*.*022***	*>0*.*999*
*NFL*	*17*.*48±2*.*81*	*18*.*09±2*.*50*	*18*.*63±4*.*45*	*>0*.*999*	*0*.*895*	*>0*.*999*
*GCL+IPL*	*50*.*76±5*.*67*	*46*.*59±5*.*93*	*45*.*88±8*.*29*	*0*.*222*	*0*.*113*	*>0*.*999*
*INL*	*25*.*54±1*.*82*	*23*.*54±3*.*66*	*22*.*40±3*.*68*	*0*.*200*	*0*.*014*	*0*.*847*
*OPL*	*16*.*94±3*.*48*	*19*.*97±8*.*37*	*18*.*36±5*.*78*	*0*.*450*	*>0*.*999*	*>0*.*999*
*HFL+ONL*	*55*.*77±7*.*59*	*49*.*37±9*.*23*	*52*.*86±8*.*97*	*0*.*101*	*0*.*972*	*0*.*720*
*MEZ*	*16*.*78±2*.*34*	*17*.*46±3*.*70*	*16*.*08±1*.*29*	*>0*.*999*	*>0*.*999*	*0*.*381*
*OS*	*23*.*15±2*.*18*	*22*.*86±1*.*48*	*22*.*26±3*.*01*	*>0*.*999*	*0*.*775*	*>0*.*999*
*IZ+RPE*	*24*.*30±2*.*60*	*23*.*85±2*.*73*	*23*.*43±3*.*32*	*>0*.*999*	*>0*.*999*	*>0*.*999*

p values were corrected by Bonferroni’s correction due to multiple testing.

Statistically significant difference at P<0.05.

### Intra-retinal thickness in the peripheral fovea

Table 2 shows the thickness of each retinal layer measured 0.5mm from the foveal center. No significant differences were discovered between the fellow eyes and control eyes as well as between amblyopic eyes and fellow eyes, for any layer in the four quadrants (p>0.052, ANCOVA).

When comparing peripheral foveal thickness between the amblyopic eyes and control eyes, we found that the total thickness of the nasal quadrant in amblyopic eyes (224.95±9.92μm)was significantly greater than in control eyes (215.10±12.36μm) (p = 0.041,ANCOVA).Exploring intra-retinal layers in the nasal quadrant, the NFL in amblyopic eyes was significantly thicker than in control eyes. The difference was 1.88μm (p = 0.01, ANCOVA). In addition, the total thickness of the inferior quadrant in amblyopic eyes (230.76±9.86μm) was significantly greater than in control eyes (219.90±12.51μm) (p = 0.022, ANCOVA). Exploring the intra-retinal layers in the inferior quadrant, the INL in amblyopic eyes was significantly thicker than in control eyes. The difference was 3.14μm (p = 0.014, ANCOVA). There were no significant differences for any layer in the temporal or superior quadrants.

After adjusting for ocular magnification, axial length was negatively correlated with nasal peripheral NFL (p = 0.04, r = -0.386) and was not correlated with any other intra-retinal layer. Refractive error was positively correlated with nasal peripheral NFL (p = 0.002, r = 0.406) and GCL+IPL (p = 0.036, r = 0.287). The severity of amblyopia had no correlation with the intra-retinal thickness of any layer in the central or peripheral fovea (p>0.079).

## Discussion

The present study evaluated the thickness of each layer of the fovea of anisometropic amblyopic eyes and compared it to fellow eyes and the eyes of children with normal vision. We found no significant differences in the central fovea between amblyopic eyes and either the fellow eyes or the control eyes for any of the four layers. There were some statistically significant comparisons between the amblyopic group and control group in the peripheral fovea. Overall, the thicknesses of the nasal and inferior quadrants in amblyopic eyes were significantly greater than in normal control eyes. These differences were due to the nasal NFL and inferior INL layer.

Although amblyopia has been shown to have a cortical and lateral geniculate basis, there remains the question of whether there is an amblyopic locus in the retina as well. Several studies have examined foveal thickness in unilateral amblyopia. First, they evaluated the average foveal thickness within a central 1mm on the macular map. Unfortunately, roughly half of these studies reported no difference in the foveas between amblyopic eyes and normal eyes.[10–12] Three years ago, our laboratory measured foveal thickness in 53 highly hyperopic, binocular, amblyopic children (mean age: 6.9 years old) and 21 esotropic amblyopic children (mean age: 9.7 years old), and we compared the results with those of normal vision control subjects. We found no significant differences for either of the two comparison.[29,30] The majority of these studies were performed with TD-OCT, which has a resolution of 10 μm axially and 20 μm in the transverse direction. However, most of the thickness differences reported by previous studies measured by TD-OCT were less than 10μm[13,14],which made the outcomes of these low-resolution instruments unreliable and the discrepancies among studies understandable.

With the development of SD-OCT, which has axial retinal resolutions of approximately 3μm, studies are no longer limited to the average thickness of the fovea. Investigators now attempt to locate each layer within the retina, considering that a slight change in one layer might not be discovered when combining all of the layers together into overall thickness. There have been a limited number of publications on the thicknesses of individual layers in the normal foveas of children. We measured the foveal thickness in our children at the very thinnest point in the center of the foveas of the amblyopic, fellow and control eyes to be 133μm. Grover et al.[31] reported normative data for adults with a mean foveal thickness of 202.3 ± 19.6μm in adults, while Turk et al.[32] and Yanniet al.[33] reported that the mean overall macular thickness in children was 326.44 ± 14.17μm and 271.2μm, respectively. Both Grover et al. and Turk et al. calculated mean thickness within the 1 mm-diameter central foveal subfield. This procedure combined the areas we designated the center and the peripheral foveas. Vajzovic[34] used a different SD-OCT system and found a central foveal thickness for children between 6 and 16 years old to be approximately 175μm, which was consistent with their histological studies and closer to our findings. Further, our estimate was similar to the report of developmental histology from Hendrickson.[35]Clearly, a system must be devised to standardize SD-OCT measurements across research platforms so that absolute thicknesses can be meaningfully compared.

Whether the fovea is involved in amblyopia remains an open question. Nishi et al.[16] accessed the thickness of the central fovea in the eyes of children with hyperopic anisometropic amblyopia, while Park et al.[18] evaluated the thickness of the central and peripheral fovea of eyes with unilateral amblyopia. Both of them found no amblyopic differences in total central foveal thickness, consistent with the current study. Although Nishiet al.[16] and Park et al.[18] showed mean central foveal thicknesses of approximately 175μm, their studies showed significantly different thicknesses for the individual layers in the central fovea (the ONL, IS and OS). Nishi et al. suggested that the differences might be due to differences in ethnicity.[16]The thicknesses measured at the absolute foveal centers in our study were thinner than in these two studies. The ages of the subjects in the three studies were very similar, so age could not account for the foveal differences. Huynh et al.[13] reported that East Asian children had thinner foveas than Western children, so we might in part attribute the thin foveas to ethnicity, but ethnicity does not explain the amblyopia results.

Bruce et al.[19] also found no differences in foveal thickness between amblyopic and fellow eyes. However, he did report that the foveal thicknesses of both eyes of amblyopes were increased compared to age-matched controls. Based on this finding, he emphasized that the lack of visually normal control subjects hid the structural differences that exist between individuals with and without amblyopia but not those between amblyopic and fellow eyes. We included an age-matched control group, but our results showed no central foveal thickness differences between the eyes of amblyopic and normal sighted control subjects. However, our results reveal that intra-retinal thickness differences at locations 0.5mm from the foveal center existed between individuals with amblyopia and visually normal control subjects, although the differences were scattered and small. Interestingly, the MEZ showed a significant difference between the non-amblyopic eyes and normal control eyes, indicating that the fellow eyes of amblyopic eyes could be changed during amblyopic development, which agreed with Bruce’s statement, although the significance was small.

When examining layers within the fovea, Nishi et al.[16] reported shorter outer segments in the central fovea of anisohypermetropic amblyopic eyes compared with fellow eyes. However, Park et al found no differences in any retinal layers in central fovea but in peripheral fovea, which agrees with our results. But Park et al[18] reported significant differences between two eyes in unilateral amblyopia in the ganglion cell layer plus inner plexiform layer, inner nuclear layer, outer plexiform layer and outer nuclear layer at several macular locations, which have not been demonstrated by the present study. The difference between studies might best be attributed to technical differences rather than anatomical differences. The four children’s amblyopia studies using SD-OCT used different SD-OCT systems, which might have resulted in different absolute thickness measurements. Other studies have reported that retinal thickness measurements differed significantly between OCT systems.[36]We believe that the greatest potential for differences could lie in the methods used to measure the layers’ thicknesses after the images were obtained. In two of the studies, [16,18] the thickness of each foveal layer was measured manually using calipers, while we and Bruce et al. [19] used different custom developed software for automatic layer segmentation. Our system showed excellent repeatability and reproducibility,[23] while the manual procedures are highly subjective and susceptible to variability. As we all know, finding differences as fine as only a few microns requires great precision, especially when the best instruments have a resolution limit of approximately 3μm. The boundaries between adjacent layers have thicknesses of at least 1 or 2 μm. If each research group chooses different dividing points between layers, a small shift in the boundary criteria can cause a sufficiently large difference in layer thickness to alter layer comparisons between subjects. Our system and Bruce et al.’s system provided a best fit curve for the surfaces of the retina, so the thinnest point in the central fovea could be precisely interpolated, and we could analyze the shape of the foveal pit. Measurement with calipers requires using a broader area than only the precise central point and would thus result in a thicker central fovea measurement and an inability to perform other fine analyses.

## Conclusion

We did not find a structural abnormality in the central foveas of children with anisometropic amblyopia. Overall, the peripheral foveal retina was thicker in the amblyopic eyes than the normal control eyes in all four quadrants, attaining significance in 2 of the 4 quadrants. These differences were due to differences in the NFL and INL layers. However, the differences were scattered, and they did not form a pattern. There have been few consistencies among studies of this issue. Thus, we recommend that future studies use spectral-domain OCT with automatic layer segmentation software that is consistent between laboratories. We should pay special attention to potentially subtle changes in the peripheral fovea. Larger samples and control subjects are still needed.

Retinal involvement in the genesis of amblyopia is a serious question. It must be answered before we can fully understand the known central visual system deficits that are assumed to cause visual deficits due to amblyopia. New instrumentation promises to clarify the influence of the amblyopic retina if we are sufficient clever to determine how to use it for this purpose.
